# Whole-Genome Sequencing: An Effective Strategy for Insertion Information Analysis of Foreign Genes in Transgenic Plants

**DOI:** 10.3389/fpls.2020.573871

**Published:** 2020-12-01

**Authors:** Xu-jing Wang, Yue Jiao, Shuo Ma, Jiang-tao Yang, Zhi-xing Wang

**Affiliations:** ^1^Biotechnology Research Institute, Chinese Academy of Agricultural Sciences/MARA Key Laboratory on Safety Assessment (Molecular) of Agri-GMO, Beijing China; ^2^Development Center for Science and Technology/MARA, Beijing, China

**Keywords:** whole-genome sequencing, molecular characterization, copy number, insertion site, flanking sequence, transgenic event

## Abstract

Molecular characterization is a key step in the risk assessment of genetically modified organisms (GMOs) for regulatory approval. Herein, we describe a method for analyzing copy number, insertion loci, and flanking sequences through whole-genome sequencing (WGS) and bioinformatics. Comprehensive molecular characterization of G2-6 transgenic rice was performed using this pipeline. The results showed that one copy of the foreign gene was inserted into rice chromosome 8. There was no vector backbone insertion but an unexpected insertion and DNA rearrangement at the 3′ end of the T-DNA. We also obtained the 5′ and 3′ flanking sequences of the T-DNA. Our results suggested that the use of a combination of WGS and bioinformatics is an effective strategy for the molecular characterization of GMOs.

## Introduction

Genome-level molecular characterization of genetically modified organisms (GMOs), including information about the copy number, location and integrity of the exogenous gene in the plant genome, and the flanking host DNA sequence of the foreign DNA fragment, is key to detecting transgenic events. Such characterization is a key step in obtaining desirable products, crucial for safety assessment and the main basis of safety supervision and detection by administrative departments. Correspondingly, some organizations have provided data requirements for molecular characterization (Food and Agricultural Organization of the United Nations., 2003; Organization for Economic Cooperation and Development, 2010; EFSA Panel on Genetically Modified Organisms, 2011).

There are different approaches to molecular characterization. Southern blotting (Southern, 1975), digital PCR (Collier et al., 2017; Sun and Joyce, 2017), and quantitative real-time PCR (qRT-PCR) (Ingham et al., 2001; Song et al., 2002; Bubner and Baldwin, 2004) are effective methods for copy number analysis. PCR-based genome walking, such as inverse PCR (Ochman et al., 1988), adaptor-mediated PCR (Huang et al., 2007), and thermal asymmetric interlaced PCR (TAIL-PCR) (Liu et al., 1995), is combined with Sanger sequencing to identify flanking sequences (Food and Agricultural Organization of the United Nations., 2003; Li et al., 2017). These techniques possess distinct characteristics and present different technical challenges. For example, Southern blotting can directly reflect the copy number of an exogenous gene in transgenic plants and has high accuracy and repeatability. However, it is labor intensive and time consuming, requires skilled operators, and does not provide sequence-level information (Zastrow-Hayes et al., 2015). PCR-based methods are not sufficient for discovering rearrangements of exogenous DNA fragments, all insertion sites, and their flanking sequences in transgenic plants with multilocus insertion and cannot identify undocumented molecular characteristics in GM crops (Wahler et al., 2013; Li et al., 2017). Moreover, it is urgently necessary to explore new strategies for molecular characterization with the development of stacked GM crops and gene-edited plants.

Given the development of next-generation sequencing (NGS) and bioinformatics tools, whole-genome sequencing (WGS) can be easily completed at a low cost in a short time and is therefore widely used in the life sciences. WGS combined with bioinformatics analysis has been used to identify the copy number, insertion site, and flanking host DNA sequence of foreign DNA fragments in transgenic Arabidopsis (Lepage et al., 2013), soybean (Kovalic et al., 2012; Guo et al., 2016; Guttikonda et al., 2016), rice (Yang et al., 2013; Park et al., 2015, 2017; Zhang et al., 2020), and maize (Cade et al., 2018). Compared with Southern blotting and PCR-based methods, the combination of WGS with *de novo* assembly and bioinformatics analysis, which has many desirable characteristics, such as a high degree of standardization, good repeatability, high automation, and high accuracy, has become a preferred approach for molecular characterization.

G2-6 transgenic rice carries the glyphosate tolerance gene *G2-aroA* and was originally obtained using Agrobacterium-mediated transformation. Southern blotting analysis revealed one copy of the foreign gene in the rice genome. The 3′-end flanking sequence of the foreign DNA fragment was not obtained by the PCR-mediated genome-walking method (Dong et al., 2017). In this study, we determined the copy number, integrity, insertion site, and flanking sequence of foreign DNA fragments and identified the presence or absence of vector backbone sequences in transgenic plants by combining WGS data and bioinformatics analysis. We intended to provide insights into the advantages of applying different sequencing platforms and sequencing depths in the molecular characterization of transgenic plants using the WGS approach.

## Materials and Methods

### Plant Material

Three samples, transgenic rice G2-6, conventional rice cultivar Zhonghua 11 (ZH11), and spike-in control (ZH11-P), were used for WGS analysis. Transgenic rice G2-6 containing the *G2-aroA* gene was generated by Agrobacterium-mediated transformation (Dong et al., 2017). ZH11 was the recipient of G2-6. ZH11-P was produced through adding one copy per genome equivalent transformation plasmid pUBIG2 DNA in ZH11. This copy frequency was determined by weight, based on a rice genome size of 389 Mb, and calculated according to the reported formula. All samples have similar genetic backgrounds. The known DNA sequences of pUBIG2 served as references for bioinformatics analyses.

### Genomic DNA Isolation and Quantification

Rice samples were planted at a greenhouse in CAAS. Five plants growing normally at the same stage (3–5 leaves) were selected randomly from G2-6 and ZH11 for the extraction of total genomic DNA. Total genomic DNA of rice leaves was extracted using a DNA extraction kit. To produce spike-in positive control samples for sequencing, the plasmid DNA was added to the genomic DNA of ZH11 at one copy per genome equivalent. DNA quantity was evaluated by 1% agarose gel electrophoresis and a NanoDrop instrument (Thermo Scientific, United States).

### WGS Using the Illumina Platform

The DNA library was prepared using an NEBNext Ultra DNA Library Prep Kit in accordance with the manufacturer’s protocol. The DNA was sheared randomly using a Covaris ultrasonicator (Covaris, Inc.), and the DNA fragments were subjected to end repair, A-tail addition, and adapter ligation. The resultant DNA was purified using an Agencourt SPRI Kit (Beckman Coulter Life Sciences, United States) to select DNA fragments with an average insert size of 350 bp. The DNA fragments with adapter molecules at the 5′ and 3′ ends were selectively enriched by PCR, and the libraries were detected using an NGS 3K/Caliper assay and quantified by qPCR.

Four DNA libraries, containing negative control ZH11 DNA library, two transgenic rice G2-6 DNA libraries (one is for 70× sequencing, another is for 30× sequencing), and a spike-in positive control DNA library, were constructed. The DNA libraries were sequenced with 150-bp paired-end sequencing on an Illumina HiSeq 4000 (Illumina, San Diego, CA, United States) instrument following the manufacturer’s protocol.

### WGS Using the PacBio Platform

Genomic DNA was fragmented using a 26-G needle. Sheared DNA fragments were subjected to DNA damage repair and purified using the BluePippin automatic nucleic acid electrophoresis and fragment recovery system and a BluePippin reagent kit to select DNA fragments longer than 20 kb. The resultant DNA was end repaired and adapter ligated using an NEBNext Ultra DNA Library Prep Kit. The DNA library was sequenced on the PacBio Sequel platform. There are two libraries constructed. One is a DNA library for G2-6 and another is for ZH11.

### Molecular Characterization Using Illumina Sequencing Data

Raw sequencing reads were filtered to obtain clean reads by removing adapter sequences and sequences with Phred scores below 20 using FastQC software. The clean reads were mapped to the pUBIG2 plasmid sequence using Burrows–Wheeler Aligner (BWA) 0.7.17 software (Li and Durbin, 2009). Reads were organized into three groups: (1) reads that fully matched the pUBIG2 plasmid sequence; (2) reads that partially matched the pUBIG2 plasmid sequence, which were derived from the location of insertion sites that spanned the junction between insertion and plant sequences; and (3) reads that did not match the pUBIG2 plasmid sequence, which were derived from the recipient genome. Reads fully or partially derived from the pUBIG2 plasmid were format conversion, arrangement, and de-duplication using Sequence Alignment/Map tools (SAMtools version 1.6) and visualized with Integrative Genomic Viewer (IGV) (Supplementary Figure S1). From the IGV results, reads that matched the plasmid sequences were used to rebuild the inserted sequence backbone residual structure and determine copy number. Reads with one end mapped to the reference sequence and the other mapped to the end of the T-DNA were collected and subjected to multiple-sequence alignment to determine the insertion site and flanking sequence using NCBI BLAST against the rice reference genome (*Oryza sativa*).

### Molecular Characterization Using PacBio Sequencing Data

Polymerase reads were separated from adapters and filtered to obtain subreads using SMRT Link v4.0 software. Then, the subreads were filtered to remove those with a minimum length less than 50 bp and a read quality below 0.8. The clean reads were mapped to the pUBIG2 plasmid sequence using Minimap2 software (Li, 2018).

### Confirmation of Insertion Loci and Flanking Sequences by PCR and Sanger Sequencing

Primers 6-3-F (CTGCACTTCAAACAAGTGTGACA), 6-3-R (ACGACGCCGTGACCACATGCAT), 6-5-F (CCGTATCGC TGTTCAAAACG), and 6-5-R (GAAATAGAGTAGATGC CGACCG) specific to the T-DNA insert and the flanking genomic DNA of G2-6 GM rice were designed. Amplification was performed under the following conditions: 98°C for 30 s, followed by 35 cycles of 98°C for 10 s, 65°C for 15 s, and 65°C for 15 s. The PCR products were sequenced by Sanger sequencing.

## Results

### Workflow for Molecular Characterization Using WGS

**FIGURE 1:**
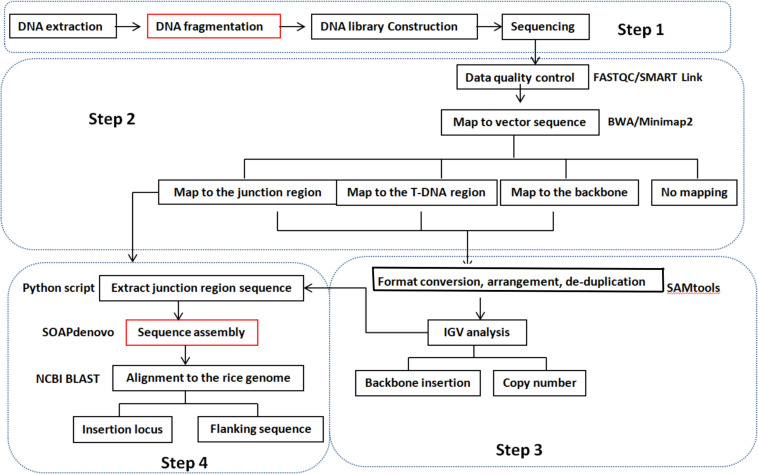
Pipeline for the molecular characterization of transgenic plants using the WGS method. Step 1: DNA library prepared according to the need of different sequencing platforms. Step 2: clean reads were obtained through FASTQC/SMART Link software from raw sequencing data. Then, clean reads were mapped to a transformation vector pUBIG2 sequence using BWA/Minimap2 software. Clean reads were clarified into four types: fully mapped to the T-DNA region, fully mapped to the backbone region, partially mapped to the T-DNA region, and reads do not map to the vector sequence. Step 3: the mapped reads were visualized by IGV software after format conversion, arrangement, and de-duplication using SAMtool. The copy number of T-DNA and backbone insertion was obtained according to the number of junction site number and coverage compared with spike-in positive control with the same sequencing depth. Step 4: reads containing the junction region sequence between T-DNA and recipient genome were achieved through the Python script process or IGV analysis. Then, the DNA fragment containing the junction sequence was obtained through sequence assemble software SOAPdenovo based on the extracted reads of the junction region and BLAST to the reference genome of the recipient. The T-DNA insertion site and flanking sequence were found according to the BLAST result. The steps noted by red frames are needed only for the Illumina sequencing platform.

We developed a pipeline for detecting copy number, insertion loci, and flanking sequences using WGS data instead of conventional detection methods. A diagram of the pipeline is shown in Figure 1. Qualified sequencing data were aligned against a transformation vector sequence using Burrows–Wheeler Aligner software with maximal exact matches (BWA-MEM) (Li and Durbin, 2009). Mapped reads were classified into three groups based on T-DNA location. The classes of reads fully mapped to the transformation vector were connected to analyze the backbone insertion and integration of T-DNA insertion. Reads with partial mapping to the transformation vector sequence will be referred to junction reads and used to determine the insertion site. The copy number was estimated by comparing the coverage of T-DNA between transgenic event and spike-in positive control sample, for example, if there are two cases of junction reads and the coverage of reads is similar with the spike-in positive control sample that means one copy number and one insertion site of foreign gene at the recipient genome. The copy number of the T-DNA and backbone insertion can be present through visualized analysis using IGV software. The reads that are mapped to the junction region of the T-DNA and rice genome were extracted using the Python script program. Flanking sequence and insertion loci can be obtained through sequence assembly using SOAPdenovo software based on the extracted junction reads and NCBI BLAST. The detailed molecular characterization of transgenic event can be acquired by above analysis process.

### WGS of G2-6 Transgenic Rice

With the Illumina sequencing platform, a total of 9.13–48.50 Gb of clean bases was obtained from the samples, accounting for 23× to 125× coverage of the rice genome. The percentage of sequencing data with Phred-like quality scores higher than 30 was greater than 90%, indicating the high quality of the sequencing data (Table 1). The 20× and 40× sequence data of G2-6 are derived by subsampling from 70× sequence data; the 30× sequence data come from another sequencing run using another DNA library.

**TABLE 1:** Statistics of Illumina sequencing quality control.

	**DNA resource**	**Data resource**	**Raw bases**	**Raw reads**	**Clean bases**	**Clean reads**	**Q20**	**Q30**	**GC content**	**Coverage^a^**
ZH11	ZH11 leaves DNA from five plants	Sequencing data of ZH11 DNA library with 30× depth	28.87G	192 509 012	28.22G	188 156 796	97.42%	93.47%	44.98%	73
ZH11-P	ZH11 leaves DNA with 1 copy plasmid DNA plants	Sequencing data of pipe-in DNA library with 30× depth	15.73G	104 873 172	15.43G	102 843 198	97.38%	93.41%	44.15%	40
G2-6-20	G2-6 leaves DNA from five plants	Extract data from 70× sequencing data	10.37G	69 196 530	9.13G	60 837 710	96.84%	91.86%	43.96%	23
G2-6-30	G2-6 leaves DNA from five plants	Sequencing data of G2-6 DNA library with 30× depth	21.82G	145 526 950	21.33G	142 186 510	97.40%	93.61%	43.16%	55
G2-6-40	G2-6 leaves DNA from five plants	Extract data from 70× sequencing data	18.89G	125 983 960	17.12G	114 144 436	96.95%	92.11%	43.84%	44
G2-6-70	G2-6 leaves DNA from five plants	Sequencing data of another G2-6 DNA library with 70× depth	53.67G	357 805 260	48.5G	323 365 034	96.73%	91.62%	43.91%	125

*^*a*^Rice genome coverage [estimated genome size: 389 Mb (International Rice Genome Sequencing Project, 2005; van Dijk et al., 2014)].*

With the PacBio sequencing platform, the length of the effective subreads was greater than 9000 bp (Table 2). The percentages of valid polymerase reads and subreads were 38.9 and 45.7%, respectively. These results indicated that the sequencing data were abundant and reliable.

**TABLE 2:** Statistics of PacBio sequencing quality control.

**Sample**	**DNA resource**	**Read_type**	**Read_bases**	**Read_number**	**Read_length (max)**	**Read_length (mean)**	**Read_length (N50)**
G2-6	G2-6 leaves DNA from five plants	Polymerase	16,486,874,422	594,173	128,681	27,747	53,327
		Insert size	8,691,175,341	594,173	121,040	14,627	19,269
		Subreads	16,454,962,990	1,296,891	121,040	12,688	15,916
ZH11	ZH11 leaves DNA from five plants	Polymerase	16,122,949,768	694,867	132,157	23,202	57,575
		Insert size	6,944,640,826	694,867	110,933	9,994	12,913
		Subreads	16,073,791,364	1,779,967	110,933	9,030	10,501

### Molecular Characterization of G2-6 Transgenic Rice Through Illumina Sequencing Data

Clean reads were mapped to the sequence of the pUBG2 vector. The reads containing the junction site sequence and vector sequence were chosen and aligned to the entire T-DNA sequence to determine the insertion site, copy number, and integrity of the insertion sequence.

**FIGURE 2:**
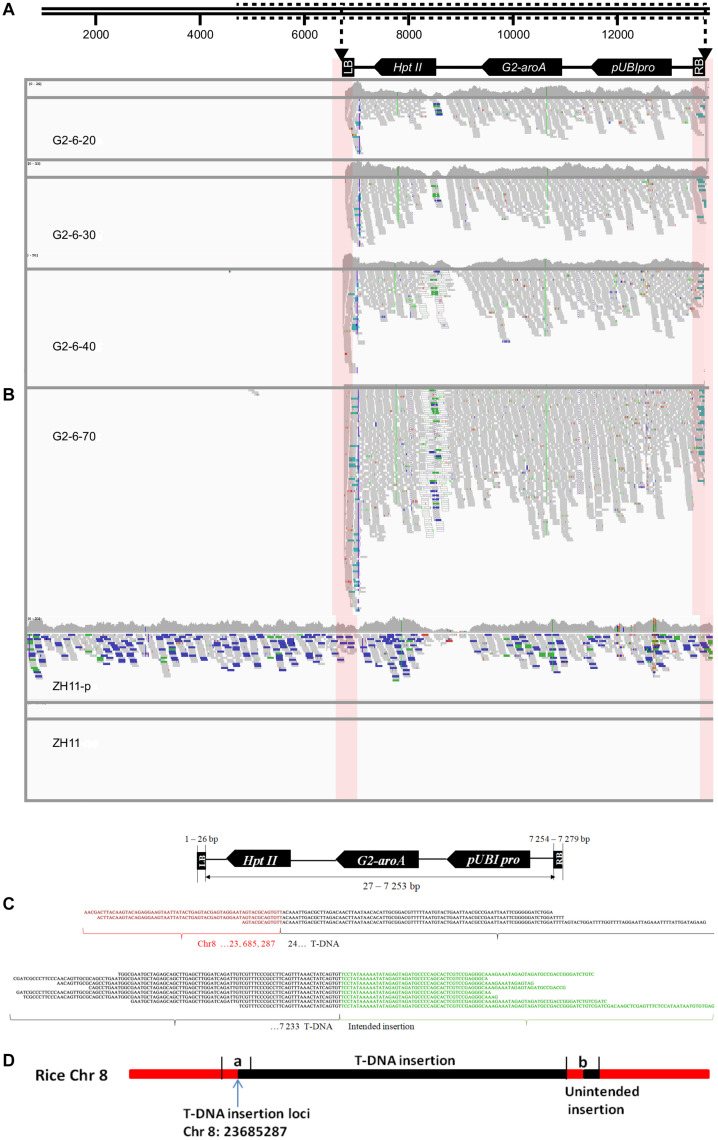
Insertion information analysis of G2-6 using the WGS method and the Illumina platform. **(A)** Illustration of the transformation vector pUBIG2 containing T-DNA. The full length of the vector was 13512 bp. The T-DNA region is located from 6295 to 61 bp. **(B)** Detailed results of IGV analysis of G2-6 for different sequencing depths and positive and negative controls. Pink boxes indicate the junction region between the T-DNA border and the rice genome. **(C)** Sequence alignments of junction-region reads (the upper panel presents the flanking sequences of the 5′ end of the T-DNA, and the lower panel presents the flanking sequences of the 3′ end of the T-DNA). Red, black, and green nucleotide sequences represent the rice genome sequence, T-DNA sequence, and intended insertion sequence, respectively. **(D)** The sketch map of T-DNA insertion information at the rice genome. The T-DNA was inserted into the rice Chr8 23685287 site. (a) T-DNA from 24 to 246 bp; (b) the partial fragment of intended insertion, the reverse and complementary sequence of T-DNA from 24 to 246 bp.

A total of 11 to 121 junction reads were obtained from the G2-6 sequencing data with different sequencing depths (Table 3). According to the IGV analysis results, the T-DNA coverage depth was the same as that for zh11-P at the same sequencing depth, but the coverage depth at the 5′ end of the T-DNA was almost 2 times that of other parts of the T-DNA (Figures 2A,B). It indicated that this T-DNA region may have a DNA rearrangement. Two junction sites were found at all sequencing depths (Figures 2B,C). No reads were mapped to the backbone region of the pUBIG2 vector sequence when the sequencing depth was 20× or 30×. However, 1 read and 3 reads were mapped to the 4427–4663-bp region of the vector backbone when the sequencing depth was 40× and 70×, respectively (Figures 2A,B). The PCR amplification results showed that this was not the true backbone insertion location.

**TABLE 3:** Statistical results for the reads mapped to different vector regions.

**Sample**	**Reads mapped to the T-DNA**	**Reads mapped to the vector backbone**	**Reads mapped to the junction site at the 3′ end of the T-DNA**	**Reads mapped to the junction site at the 5′ end of the T-DNA**
ZH11	0	0	0	0
ZH11-P	972	2784	10	10
G2-6-20	514	0	4	7
G2-6-30	993	0	8	8
G2-6-40	829	1	6	7
G2-6-70	2885	3	36	85

Junction reads obtained from G2-6 transgenic rice (sequencing depth: 70×) were *de novo* assembled using SOAPdenovo software. Junction sequences of 604 bp were obtained from the 5′ end of the T-DNA insertion. Through alignment and BLAST analysis, the 5′-end junction sequence was found to contain a 278-bp rice genome sequence, which was properly mapped to the 23685010–23685287-bp region of rice chromosome 8, and a 326-bp vector sequence, which was properly mapped to the T-DNA region from 24 to 350 bp. Junction sequences of 365 bp were obtained from the 3′ end of the T-DNA insertion. Of this sequence, 123 bp was the 3′ end of the T-DNA, 223 bp was the reverse and complementary sequence of the 5′ end of the T-DNA from 24 to 246 bp (Figure 2D), and no rice genome DNA was obtained. This proved the IGV analysis result that there was a DNA rearrangement of DNA fragment of the 5′ end of the T-DNA and junction sequence.

The above result means that T-DNA was inserted into the 23685287-bp site of chromosome 8 and there was an unintended insertion at the 3′ end.

### Molecular Characterization of G2-6 Transgenic Rice Through PacBio Sequencing Data

**FIGURE 3:**
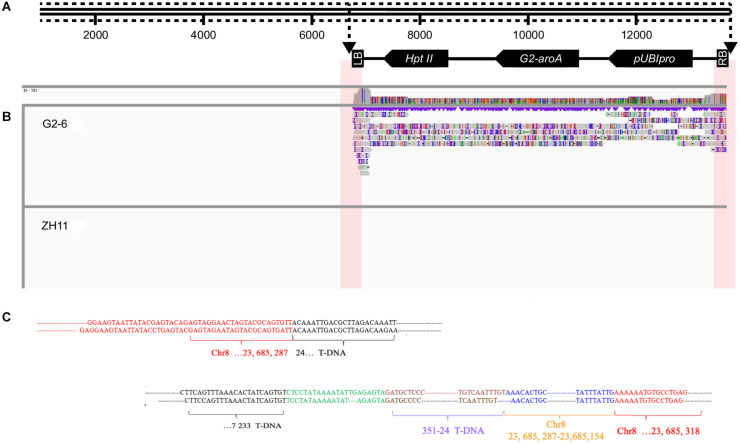
Detailed results of IGV analysis of G2-6 using PacBio sequencing data. **(A)** Illustration of the transformation vector pUBIG2 containing T-DNA. The full length of the vector was 13512 bp. The T-DNA region is located from 6295 to 61 bp. **(B)** Detailed results of IGV analysis of G2-6 using PacBio sequencing data. **(C)** Sequence alignments of junction-region reads. The upper panel presents the flanking sequences of the 5′ end of the T-DNA. The lower panel presents the flanking sequences of the 3′ end of the T-DNA and intended insertion.

Subreads were mapped to the sequence of the pUBIG2 vector using smart software. The reads containing the junction site sequence and vector sequence were chosen for further analysis. Eleven and seven junction reads were obtained through analysis. The IGV analysis results revealed two junction sites (Figure 3A).

Two reads were selected from the 5′ junction site for alignment with the rice reference genome. A 1973-bp junction sequence was obtained from the 5′ junction site. This DNA fragment contained a 1390-bp T-DNA sequence, which was properly mapped to the T-DNA region from 24 to 1408 bp, and a 583-bp rice genome sequence, which was properly mapped to the 23684706–23685287-bp region of rice chromosome 8. A deletion of 23 bp was observed in the T-DNA region (Figure 3B). This result is consistent with the results from the Illumina sequencing data.

Two reads from the 3′ junction site were selected for alignment with the rice reference genome. A 3720-bp junction sequence was obtained. The DNA fragment contained a 532-bp T-DNA sequence, which was properly mapped to the T-DNA region from 6701 to 7233 bp; a 327-bp sequence, which was the reverse and complementary sequence of the T-DNA from the 24 to 351-bp region; a 134-bp sequence, which was the reverse and complementary sequence of the region from 23685154 to 23685287 bp on rice chromosome 8; and a 2639-bp sequence, which was properly mapped to the 23685318 to 23687941-bp region of rice chromosome 8. A deletion of 46 bp was observed at the right border (RB) of the T-DNA (Figure 3C).

**FIGURE 4:**
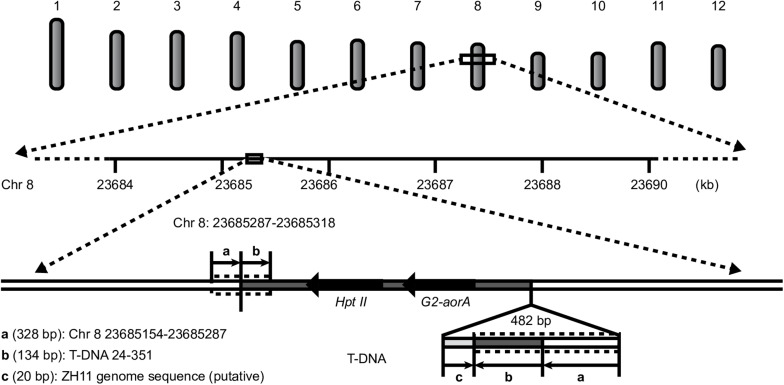
T-DNA insertion information for G2-6.

All the results showed a single copy of the T-DNA inserted into the rice genome, a 23-bp deletion at the left border (LB), a 49-bp deletion at the RB, and no backbone insertion. The insertion locus was located on chromosome 8 from 23685287 to 23685318 bp, with a 31-bp deletion. An unexpected insertion sequence of 549 bp was found between the RB of the T-DNA and the 23685318-bp site of rice chromosome 8, which occurred during plant transformation (Figure 4).

### Confirmation of Insertion Sites and Flanking Sequences by PCR Amplification and Sanger Sequencing

To validate the above results regarding the location of the T-DNA in G2-6, we designed primers according to the flanking host DNA sequence and T-DNA sequence. The PCR amplification results showed that the target DNA band could be obtained when using G2-6 genomic DNA as the template (Supplementary Figure S2A). The Sanger sequencing results verified that the T-DNA insertion locus of G2-6 could be successfully characterized using WGS. However, the Sanger sequencing results showed that the unintended insertion sequence is a 482-bp DNA fragment containing a 328-bp reverse and complementary sequence of the T-DNA 24–351-bp region, a 134-bp reverse and complementary sequence of the region on rice chromosome 8 from 23685154 to 23685287 bp, and a 20-bp sequence that may come from ZH11 and be shorter than that detected with PacBio sequencing. The reason may be that PacBio sequencing results in meaningless base insertions, such as TTTTTT (Figure 4 and Supplementary Figure S2B,C).

## Discussion

With the development of high-throughput sequencing technology, WGS has become easier and more rapid. WGS is a powerful tool for detecting the rearrangement of DNA and copy number variation. Recently, it was proven that the combination of WGS and bioinformatics is a selective strategy for molecular characterization of transgenic events. Different technology pipelines for the analysis of copy number, insertion sites, or flanking sequences have been reported (Kovalic et al., 2012; Lepage et al., 2013; Zastrow-Hayes et al., 2015; Guo et al., 2016; Park et al., 2017; Zhang et al., 2020). In this study, we determined the copy number, insertion site, and host genome flanking sequence and detected vector backbone insertion and unintended integration in G2-6 using our pipeline. We found one copy of the T-DNA insertion on chromosome 8 in G2-6 rice with a 482-bp unintended insertion sequence and no vector backbone residual insertion. The results showed that WGS coupled with the developed pipeline is an effective strategy for the detailed molecular characterization of transgenic events.

A sufficient amount of high-quality sequencing data is important for the molecular characterization of transgenic events when using WGS combined with a bioinformatics approach. In this study, we assessed the effect of sequencing depth (20×, 30×, 40×, and 70×) on molecular characterization. We found that the results were related to the copy number obtained using WGS data at different sequencing depths. However, for insertion site and flanking sequence analysis, a high sequencing depth is needed to obtain a sufficient number of reads containing junction site information for reliable results (Table 3). For G2-6, we could extract 5′-end junction site reads only when the sequencing depth was 70×. This result is consistent with those in some previous publications, suggesting that a high sequencing depth is needed for the precise molecular characterization of transgenic events (Wang et al., 2008; Ajay et al., 2011). It is worth mentioning that random sequencing of reads and uneven data output are exited and inevitable during the NGS process. The DNA library quality, sequencing batch, and instrument status will affect the quality and quantity of sequencing data. In this study, data statistics showed that the sequencing data of 30× was higher than that of 40×, which may be caused by the different batches of sequencing and the DNA library used. Therefore, in order to obtain more accurate data, the test samples and control samples should be sequenced at the same time.

As one of highest-throughput platforms, Illumina offers the lowest per-base cost (van Dijk et al., 2014). However, the limitation of a short read length makes Illumina sequencing poorly suited to some molecular characterization analyses of transgenic events, in which the foreign DNA fragment is inserted into the region with the repeated sequence, leading to an unintended insertion sequence or DNA rearrangement. PacBio SMRT sequencing offers longer read lengths and an alternative to overcome this limitation (Rhoads and Au, 2015). Here, we obtained only the 5′-end flanking sequence of T-DNA using Illumina sequencing data because of the unintended insertion at the 3′ end. Then, we obtained the 3′-end flanking sequence, the insertion site, and unintended insertion information through PacBio sequencing data. Therefore, we think that PacBio sequencing is more suitable for complex transgenic events, such as multicopy insertion at the same locus, stacked transgenic events, and gene editing in crops. Notably, the error rate of PacBio sequencing is relatively high (approximately 11–15%) (Korlach, 2015). Therefore, when analyzing flanking sequences, Sanger sequencing should be combined with PacBio sequencing technology to ensure the accuracy of the result. On the other hand, the weaknesses of PacBio, i.e., the lower throughput and higher cost compared with those of Illumina sequencing, also restrict its application (Rhoads and Au, 2015).

Often, some reads are mapped to the backbone of vector plasmids when obtaining backbone insertion information using WGS. These reads may come from the true insertion of the backbone, a homologous sequence of the backbone in the host genome, or contamination during DNA library preparation. The source can be determined through coverage level analysis and alignment with the host genome (Cade et al., 2018). Three reads in the G2-6 Illumina sequencing data were mapped to the backbone of the pUBIG2 plasmid sequence when the sequencing depth was 70×. These reads aligned to the 4427–4663-bp region of the pUBIG2 backbone sequence. The coverage level of these reads was much lower than that of the T-DNA region. BLAST alignment demonstrated that they are not homologous sequences in the rice genome. Combining this result with the results that no reads were mapped to the backbone region of pUBIG2 when the sequencing depth was 20×, 30×, and 40×, we think that these reads are false positives caused by contamination, which is very common and difficult to completely avoid. This contamination was confirmed by PCR amplification using primers designed for the corresponding backbone region sequence.

## Data Availability Statement

The raw sequence data reported in this article have been deposited in the Genome Sequence Archive (Genomics, Proteomics & Bioinformatics 2017) in National Genomics Data Center (Nucleic Acids Res 2020), Beijing Institute of Genomics (China National Center for Bioinformation), Chinese Academy of Sciences, under accession number CRA003306 (for G2-6) and CRA003307 (for Zhonghua 11) that are publicly accessible at https://bigd.big.ac.cn/gsa. Orders, code, detailed pipeline documentation and parameter settings have been privy on persistent public platform GitHub (https://github.com/GUOWEIJUN/NGS).

## Author Contributions

ZW and XW conceived and designed the experiments. SM performed the experiments. XW and YJ analyzed the data and wrote the manuscript. JY improved the language. All authors contributed to the article and approved the submitted version.

## Conflict of Interest

The authors declare that the research was conducted in the absence of any commercial or financial relationships that could be construed as a potential conflict of interest.

## Supplementary Material

Supplementary Figure 1 Orders of bioinformatic software used in this study.

Supplementary Figure 2 Confirmation of insertion sites and flanking sequences by PCR amplification and Sanger sequencing. **(A)** PCR amplification of 5′ and 3′ flanking sequences. M present 2 kb DNA marker; 1 present 5′ flanking sequences amplification of G2-6; 2 present 3′ flanking sequences amplification of G2-6; 3 present negative control ZH11 for 5′ flanking sequences amplification of G2-6; 4 present negative control ZH11 for 3′ flanking sequences amplification of G2-6. **(B)** Alignment of 5′ flanking sequence obtained by PacBIo sequencing and Sanger sequencing; **(C)** Alignment of 3′ flanking sequence obtained by PacBIo sequencing and Sanger sequencing.
