# Supplementary figures and images for: Whole-Genome Sequencing: An Effective Strategy for Insertion Information Analysis of Foreign Genes in Transgenic Plants
Source: Front Plant Sci. 2020 Dec 1;11:573871. doi: 10.3389/fpls.2020.573871 (PMC7736074; doi:10.3389/fpls.2020.573871)

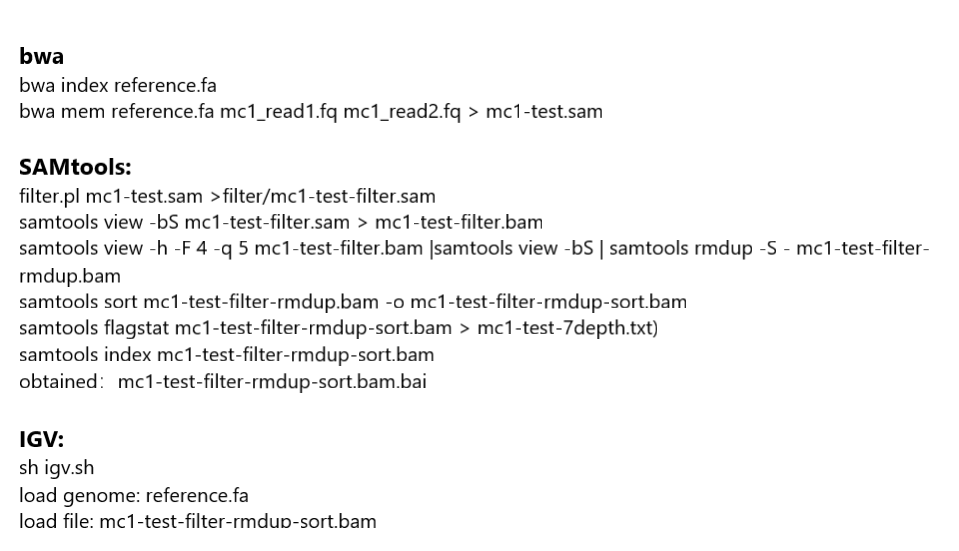

Supplement: Supplementary Figure 1 — Orders of bioinformatic software used in this study. [file Image_1.TIFF]

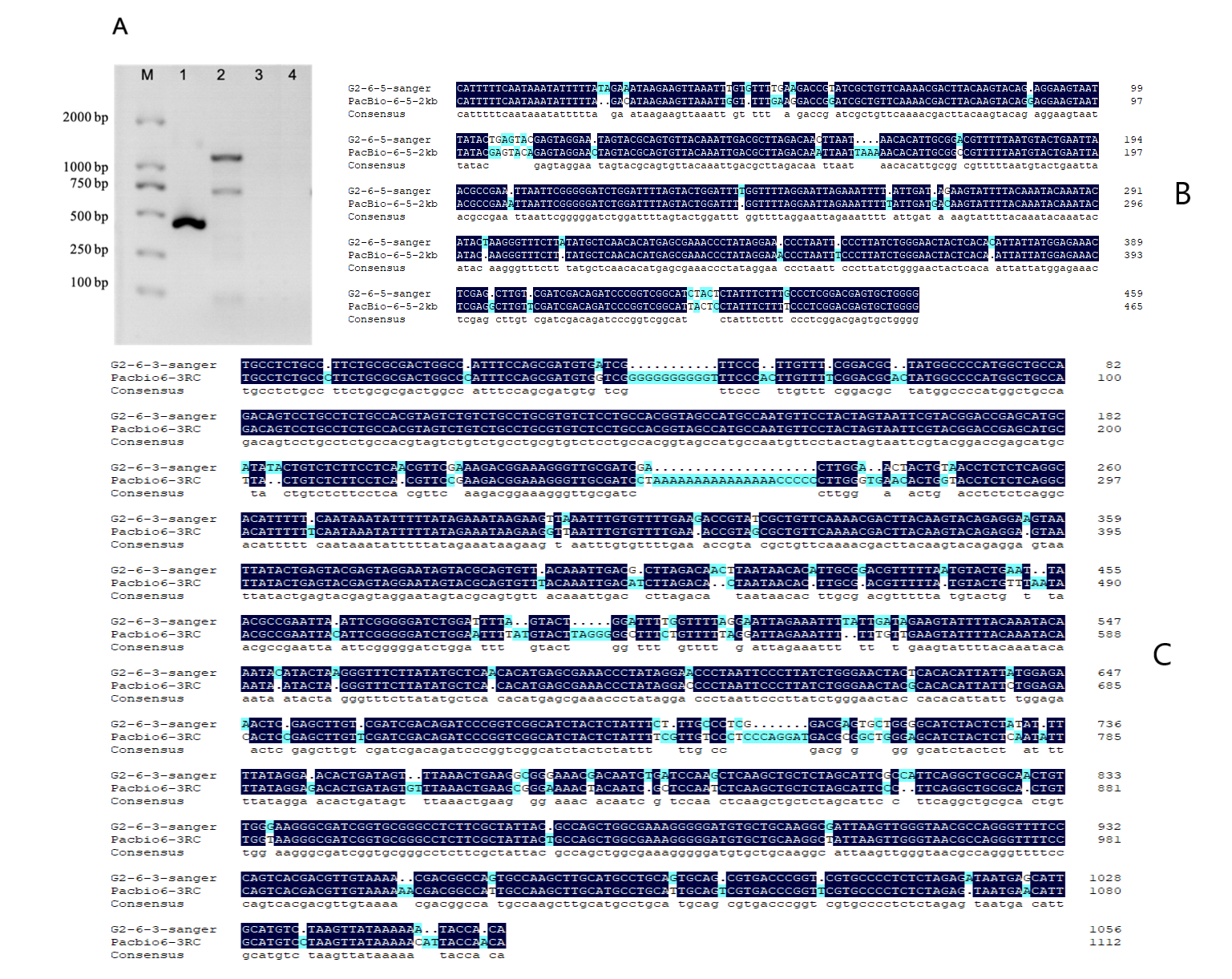

Supplement: Supplementary Figure 2 — Confirmation of insertion sites and flanking sequences by PCR amplification and Sanger sequencing. (A) PCR amplification of 5′ and 3′ flanking sequences. M present 2 kb DNA marker; 1 present 5′ flanking sequences amplification of G2-6; 2 present 3′ flanking sequences amplification of G2-6; 3 present negative control ZH11 for 5′ flanking sequences amplification of G2-6; 4 present negative control ZH11 for 3′ flanking sequences amplification of G2-6. (B) Alignment of 5′ flanking sequence obtained by PacBIo sequencing and Sanger sequencing; (C) Alignment of 3′ flanking sequence obtained by PacBIo sequencing and Sanger sequencing. [file Image_2.TIFF]
